# Immunomodulatory Effects of a Bioactive Compound Isolated from *Dryopteris crassirhizoma* on the Grass Carp *Ctenopharyngodon idella*

**DOI:** 10.1155/2016/3068913

**Published:** 2016-05-15

**Authors:** Cheng Chi, Sib Sankar Giri, Jin Woo Jun, Hyoun Joong Kim, Saekil Yun, Sang Guen Kim, Se Chang Park

**Affiliations:** Laboratory of Aquatic Biomedicine, College of Veterinary Medicine and Research Institute for Veterinary Science, Seoul National University, Seoul 151742, Republic of Korea

## Abstract

In the present study, we investigated effects of compound kaempferol 3-a-L-(4-O-acetyl)rhamnopyranoside-7-a-L-rhamnopyranoside (SA) isolated from* Dryopteris crassirhizoma* during immune-related gene expression in* Ctenopharyngodon idella* head kidney macrophages (CIHKM). The expression of immune-related genes (*IL-1β*,* TNF-α*,* MyD88*, and* Mx1*) were investigated using real-time PCR at 2 h, 8 h, 12 h, and 24 h after incubation with 1, 10, and 50 *μ*g mL^−1^ of SA. Furthermore, fish were injected intraperitoneally with 100 *μ*L of SA, and immune parameters such as lysozyme activity, complement C3, SOD, phagocytic activity, and IgM level were examined at 1, 2, and 3 weeks after injection. The differential expression of cytokines was observed after exposure to SA.* IL-1β* genes displayed significant expression at 2 and 8 h after exposure to 1–10 *μ*g mL^−1^ of SA. SA also induced gene expression of cytokines such as* MyD88*,* Mx1*, and* TNF-α*. Furthermore, enhanced immune parameters in grass carp confirmed the immunomodulatory activity of SA. Interestingly, this compound has no toxic effect on CIHKM cells as tested by MTT assay. In addition, fish immunised with 10 *μ*g mL^−1^ of SA exhibited maximum resistance against* Aeromonas hydrophila* infection. These results suggest that SA has the potential to stimulate immune responses in grass carp.

## 1. Introduction

Aquaculture is the fastest-growing sector of the animal food production industry [1], and in the last five decades, aquaculture production has grown considerably and production reached 62 million tons in 2011 [2]. However, world aquaculture production is vulnerable, and culture intensification could result in partial or total loss of production because of an increase in disease outbreaks, including infections from parasite, pathogenic bacteria, fungi, and virus diseases [3]. Generally, chemotherapeutics, vaccines, and antibiotics are used for disease control in aquaculture. However, indiscriminate use of antibiotics has resulted in the development of bacterial-resistant strains. For example, trichlorfon or praziquantel in bath treatments for parasites has disadvantages, including resistance development, potential hazards to animal health, and environmental pollution [4]. Furthermore, single vaccine application is only effective against one type of pathogen, and the vaccination of juvenile fish is labour intensive and expensive. Therefore, application of natural immunostimulants could be a viable alternative to ensure the sustainability of aquaculture. Fish primarily depend on innate immunity, and immunostimulants play a major role in enhancing innate immune responses.

Plant extracts have been reported to promote growth, stimulate appetite, and have antiparasite and antibacterial properties, as well as acting as immunostimulant in fish cultures. Some of herbal immunostimulants are alkaloids, terpenoids, tannins, saponins, glycosides, flavonoids, phenolics, steroids, or essential oils [5, 6].* Dryopteris crassirhizoma*, a semievergreen pteridophyte, is widely distributed in Japan, Korea, and China. Various plant extracts have a number of pharmacological activities, including antioxidant, tumouricidal, and fatty acid synthase inhibitory activities [7–9]. Furthermore, bioactivities of* D. crassirhizoma* are mainly caused by the presence of phloroglucinol, polyphenols, and flavonoid [10]. The active compound sutchuenoside A (SA) isolated from* D. crassirhizoma* has been shown to exhibit vermifuge activity against* Dactylogyrus intermedius* in goldfish* Carassius auratus* [11]. However, research on the immune function of bioactive components from* D. crassirhizoma* in fish is lacking.

Cytokines are low molecular weight proteins, and they include interleukins (IL), interferons (IFN), chemokines, monokines, and certain growth factors [12]. Among these cytokines, interleukin-1*β* (IL-1*β*), tumour necrosis factor-*α* (TNF-*α*), and interleukin-8 (IL-8) play a significant role in the recognition, initiation, and regulation of the inflammatory process and serve as an important component of innate immunity [13]. Moreover, myxovirus resistance-1 (Mx1) protein facilitates defense against a diverse range of viruses [14], and myeloid differentiation factor 88 (MyD88) is an important adaptor molecule in the Toll-like receptor (TLR) signalling pathway and plays an important role in host defense against bacterial infections [15]. The numerous roles of cytokines in fish immunity have been previously established. Because cytokines are important regulators of the immune system, investigating their function may provide significant information for the development of vaccines and immunostimulants for fish. Therefore, the goal of the present study was to determine if the compound sutchuenoside A, which was isolated from* D. crassirhizoma*, could influence immune-related gene expression in* C. idella* kidney cells* in vitro* using a quantitative real-time PCR (qRT-PCR) based method. Furthermore, the effect of SA on immune responses and disease resistance in grass carp was investigated to determine its potential as an immunostimulant.

## 2. Materials and Methods

### 2.1. Isolation of Bioactive Compound from* Dryopteris crassirhizoma*

In our previous study, we demonstrated that kaempferol 3-a-L-(4-O-acetyl) rhamnopyranoside-7-a-L-rhamnopyranoside (sutchuenoside A, SA) isolated from* D. crassirhizoma* exhibited anthelmintic activity against* D. intermedius* in goldfish. The compound was isolated and stored as we described [11]. Dried rhizomes (8 kg) were ground into a coarse powder using a mechanical pulveriser and were ultrasound-extracted with methanol (MeOH) (10 L × 4 times) at 60°C for 40 min. The extract was filtered, concentrated under reduced pressure in a vacuum rotary evaporate, and desiccated to yield 508 g of methanol extract. The MeOH extract was then partitioned using petroleum ether (60–90°C) and ethyl acetate (EtOAc). The concentrated EtOAc phase was applied to silica gel column chromatography using chloroform–methanol (gradient; 1 : 0 → 0 : 1;  v/v) solvent mixtures and three fractions were obtained (A–C). All fractions were monitored and combined based on thin-layer chromatography. Compound SA was isolated from fraction B as a yellowish powder by repeated silica gel column chromatography using the chloroform–methanol mixture as the eluent. Stock solutions were prepared by dissolving fractions and pure compounds in dimethyl sulfoxide (DMSO) at the concentration of 100 mg mL^−1^.

### 2.2. Experimental Fish

Healthy grass carp (average body weight: 103.2 ± 3.4 g) was procured from a local fish farm and acclimatised to laboratory conditions (dissolved oxygen: 5.50 ± 0.68 mg L^−1^; pH: 7.2 ± 0.5; nitrites: 0.022 ± 0.01 mg L^−1^; ammonia: 0.14 ± 0.05 mg L^−1^) for two weeks in 500-L quarantine tanks at 23 ± 1°C. Fish were fed a basal diet [16] during the acclimatisation period. Approximately 20% of the water in all tanks was exchanged daily, and 100% of the water was exchanged once a week. Basic physiochemical parameters of the water were measured every week.

### 2.3. *In Vitro* Study

#### 2.3.1. Isolation of* C. idella* Head Kidney Macrophage Cells and Treatment

Isolation and cultures of* C. idella* headkidney macrophage (CIHKM) cells were performed as described by Secombes [17]. Cell viability was assessed using the trypan blue (Sigma-Aldrich, USA) exclusion test, and cell number was adjusted to 1 × 10^7^ cells mL^−1^. CIHKM cells were cultured in an incubator at 28°C within sterile 6-well tissue culture plates containing 5.0 mL Minimum Essential Medium (MEM) supplemented with 10% inactivated foetal calf serum (GIBCO/BRL). For isolated compound treatment, 1 × 10^7^ cells were treated with SA (100 *μ*L) at three different concentrations of 1, 10, and 50 *μ*g mL^−1^ for 2, 8, 12, and 24 h at 28°C. For the control, CIHKM cells were incubated with 100 *μ*L of DMSO. Incubated cells were harvested at the time points mentioned above for RNA extraction. All the tests were performed in triplicate.

#### 2.3.2. RNA Extraction and Reverse Transcription

Total RNA was extracted from CIHKM cells using TRIzol Reagent (Cwbio, China). The quality and purity of RNA were assessed by spectrophotometry, and 260 : 280 ratios were 1.8–2.0. Afterwards, genomic DNA contamination was removed by the treatment with DNase I (Promega, Madison, WI, USA). DNA was then synthesised using the PrimeScript*™* RT reagent Kit (TaKaRa, Osto, Japan) following the manufacturer's instructions. The resulting cDNA was stored at −80°C.

#### 2.3.3. Real-Time Quantitative PCR Analyses of Gene Expression

The analysis of expression of immune-related genes* MyD88*,* IL-1β*,* TNF-α*, and* Mx1* was carried out using real-time quantitative PCR (Qiagen, Germany). All the qRT-PCR reactions were performed using the SYBR Premix Ex Taq*™* Perfect Real-Time Kit (TaKaRa, Osto, Japan) and carried out in the Qiagen Rotor-Gene Q Real-Time PCR Detection System (Qiagen, Germany). The *β-actin* gene was used as a house-keeping gene. The PCR primer sequences used for qRT-PCR are listed in Table 1. The reaction mixture included 10 *μ*L of SYBR Premix Ex Taq*™*, 1 *μ*L of forward and reverse primer (10 mM), and 1 *μ*L of cDNA and was filled with ultra-pure water to a final total volume of 20 *μ*L. The reaction conditions and cycle index were 95°C for 10 min followed by 40 cycles of 95°C for 15 s, 56°C for 30 s, and 72°C for 30 s. Negative control without cDNA was included in each assay. The primers used in this study were specific to grass carp; the sequences were obtained from published literature [18]. After the amplification phase, melting curve analysis was conducted to eliminate the possibility of nonspecific amplification or primer dimer formation. A standard curve was created from serial dilutions of sample cDNA. A standard curve was drawn by plotting the natural log of the threshold cycle (Ct) against the number of molecules. Standard curve of each gene was run in duplicate and three times for obtaining reliable amplification efficiency. The correlation coefficients (*R* ^2^) of all standard curves were >0.99 and the amplification efficiency was between 90 and 110%. The relative expression ratios of target genes in the treatment group versus those in the control group were calculated according to the following formula: fold changes = 2^−ΔΔCt^, where ΔΔCt = (Ct [treatment group] − Ct [treatment *β-actin*]) − (Ct [control group] − Ct [control *β-actin*]) [19]. In all cases, each three replicates of the PCR were performed.

#### 2.3.4. Cell Viability Assay

The cell viability assay was performed using the Cell Counting Kit 8 (Dojindo, Tokyo, Japan), according to the manufacturer's protocol, to measure the effect of SA on the viability of CIHKM cells. In each group, 2 × 10^4^ − 5 × 10^4^ cells were incubated in a well of a 96-well plate containing 200 *μ*L medium (MEM with 10% foetal calf serum) at 28°C in 5% CO_2_ atmosphere for 24 h. After the culture, the media in all the wells were replaced with 200 *μ*L fresh media containing SA in the concentrations of 0, 1, 10, and 50 *μ*g mL^−1^, respectively. After 2, 12, 24, 48, 72, and 96 h of incubation, solutions were replaced by 180 *μ*L DMEM without serum, and then 20 *μ*L of sterile filtered MTT (Sigma) solution in phosphate-buffered saline (PBS) at pH 7.4 (5 mg mL^−1^) was added to each well. The final MTT concentration was 0.5 mg mL^−1^ for 4 h; the unreacted dye was then removed, and the insoluble formazan crystals were dissolved in 200 *μ*L per well dimethylsulphoxide (DMSO) and shaken for 5 min. The OD value of the resulting solution was measured by a multi-well scanning spectrophotometer (ELISA reader) at 570 nm, and the culture medium without cells was considered a blank. The relative cell viability were calculated by absorbance [*A*] test/[*A*] control × 100%, where the control group contained culture medium without SA [18]. All the tests were performed in triplicate.

### 2.4. *In Vivo* Investigation

#### 2.4.1. Experimental Design

After acclimatising to culture conditions for a period of 14 days, fish were randomly divided into four groups. Each group consisted of 25 fish with three replicates (i.e., 25 fish × 3 tanks = 75 fish per group). The fish in the control group were injected intraperitoneally (i.p.) with 100 *μ*L of DMSO and the other three experimental groups were immunised i.p. with 100 *μ*L of DMSO containing either 1, 10, or 50 *μ*g mL^−1^ of SA. Fish were maintained for observation for 21 days and fed a basal diet twice a day (09:00 and 17:00 h). The composition of the basal diet is shown in Table 2.

#### 2.4.2. Blood Samples for Immunological Measurement

Three fish were randomly collected from each tank at the end of 1st, 2nd, and 3rd weeks of immunisation to measure immunological parameters. Thus, 9 fish (3 fish × 3 replicates = 9 fish) were collected from each group for immunological assays. Fish were euthanised with an over dose of MS222. Blood was sampled by caudal venipuncture using a 1-mL syringe and immediately used for leucocytes isolation as description by Cheng et al. [20]. Harvested cells were adjusted to 1 × 10^7^ cells mL^−1^ by adding an appropriate volume of RPMI 1640 (Sigma-Aldrich, USA) for cellular immunological parameter assays. Another 0.5 mL of blood from each replicate was centrifuged at 3000 ×g for 10 min at 4°C to collect serum and was then stored at −20°C until use.

#### 2.4.3. Immunological Parameters Analysis

*Lysozyme Activity*. Lysozyme activity was measured following the turbidimetric method described by Ellis [21]. A unit of lysozyme activity was defined as the amount of serum lysozyme that caused a decrease in absorbency of 0.001 min^−1^ at 530 nm.

*Complement C3 Assay*. The serum complement C3 level was assayed using the complement C3 assay Kit (Jiancheng, Nanjing, Jiangsu, China) following the manufacturer's instructions [22]. The OD was measured at 340 nm. Methods for complement C3 level analysis included the measurement of the increase in turbidity following the immunity response of complement C3 and the increase in its antibody [23, 24]. Results are presented as complement C3 mg mL^−1^.

*Phagocytic Activity*. Phagocytic activity (PA) of leucocytes was evaluated by the method of Ai et al. [25] with slight modifications. A 100 *μ*L of 1 × 10^7^ cells mL^−1^ suspension of leucocytes was placed on a sterile glass slide and allowed to attach at 25°C for 30 min. Following attachment, 100 *μ*L of 1 × 10^8^ cells mL^−1^ yeast suspension was added to the cell monolayer. The glass slides were incubated at 25°C for 45 min and then were washed with PBS (pH = 6.2) three times to remove uningested yeasts and unattached leucocyte cells. Finally, the slides were fixed with ethanol, redried, and stained with Giemsa. The number of phagocytic cells per 100 adherent cells was microscopically determined. PA was calculated using the formula:

(1) $$\begin{array}{c} \text{PA}=\left(\;\frac{\text{phagocytic leucocytes}}{\text{total leucocytes}}\;\right)\times 100 \end{array}$$

*Superoxide Dismutase Activity*. SOD activity was determined with SOD kits (Jiancheng, Nanjing, Jiangsu, China) following the manufacturer's instructions [26]. The OD was measured at 550 nm. One unit of SOD was defined as the amount required for inhibiting the rate of xanthine reduction by 50% in a one mL reaction system. Specific SOD activity was expressed as SOD units per mL of serum [27].

*Serum Immunoglobulin (IgM) Level*. Serum IgM levels were assayed by enzymatic procedures utilising an automatic biochemical analyser (Hitachi 7180, Tokyo, Japan) [26]. Total IgM was expressed as unit mg mL^−1^.

### 2.5. Challenge Study

After 21 days of immunisation, 30 fish (10 × 3 = 30) from each group were injected with 0.2 mL of PBS containing 1 × 10^7^ of live* Aeromonas hydrophila* (strain SG 322). Pathogenicity and dose of* A. hydrophila* (strain SG 322) were determined earlier [16]. Another group of 30 fish (fed basal diet during feeding trial) were injected with 0.2 mL of PBS and considered as negative control. The challenged fish were observed for two weeks, and mortality was recorded.

### 2.6. Statistical Analysis

One-way analysis of variance (ANOVA) was used to analyse the data. Multiple comparisons were performed with Tukey's test to analyse differences between treatments. All statistical analyses were performed using the OriginPro software (version 8; OriginLab Corporation, Northampton, USA). The level of significance was set at *P* < 0.05. The results are expressed as mean ± S.E.M.

## 3. Results

### 3.1. Challenge Study

**Figure 1:**
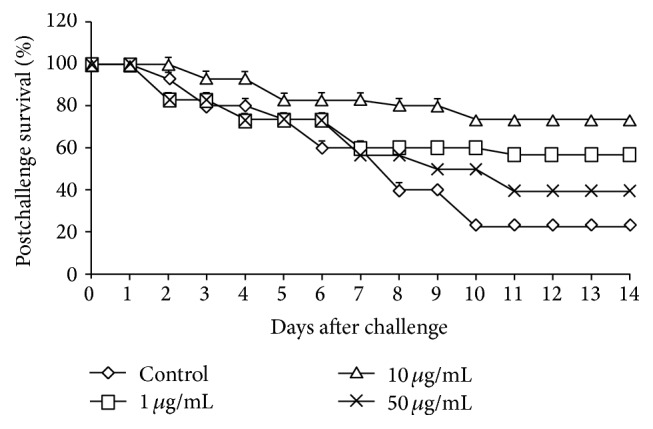
Postchallenge survival after artificial challenging with* A. hydrophila* in* C. idella* injected intraperitoneally on different assay days after infection with SA. Bars represent the mean values ± S.E.M (*n* = 3).

Two weeks of immunisation with SA enhanced the resistance of fish to* A. hydrophila* infection (Figure 1). The highest postchallenge survival (73.33%) was recorded in the fish group immunised with 10 *μ*g mL^−1^ of SA, whereas the lowest postchallenge survival rate (23.33%) was observed in the control group. Fish immunised with 1 *μ*g mL^−1^ or 50 *μ*g mL^−1^ of SA exhibited survival rates of 56.66% and 40%, respectively. No mortality was observed in the group injected with PBS only. Typical symptoms of haemorrhagic septicaemia were observed in moribund or dead fish. Colonies of* A. hydrophila* were isolated from dead fish.

### 3.2. *In Vitro* Effect of SA on the Viability of CIHKM Cells

**Figure 2:**
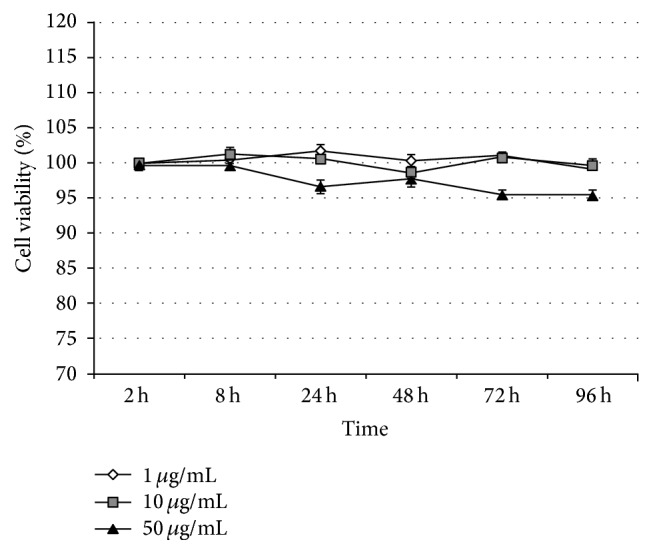
Effects of a bioactive compound SA isolated from* D. crassirhizoma* on* C. idella* head kidney macrophages (CIHKM) cells viability measured by MTT assay after 2, 8, 24, 48, 72, and 96 h incubation at 28°C. Bars represent the mean ± S.E.M (*n* = 3).

Only a 4.6% decrease (*P* > 0.05) in cell viability was observed in the CIHKM cells treated with 50 *μ*g mL^−1^ of SA for 96 h compared to that of the control. However, 1 and 10 *μ*g mL^−1^ of SA had no cytotoxic effects on CIHKM cells at any time points during the assay (Figure 2).

### 3.3. *In Vitro* Effect of SA on the Expression of Immune-Related Genes in CIHKM Cells

**Figure 3:**
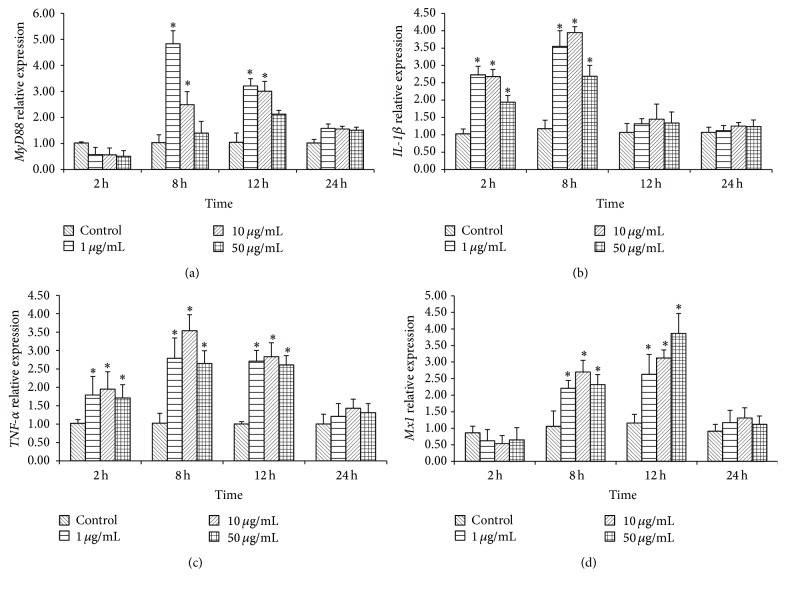
Relative expression of immune-related genes at different time points in* C. idella* head kidney macrophages (CIHKM) cells stimulated with SA. (a) Expression of* MyD88* gene; (b) expression of* IL-1β* gene; (c) expression of* TNF-α* gene; (d) expression of* Mx1* gene. Bars represent the mean ± S.E.M (*n* = 3). Significant expression levels in SA stimulated cells compared to unstimulated control are indicated by an asterisk (*∗*) at that time point (*P* < 0.05).

Expressions of immune-related genes (*MyD88*,* IL-1β*,* TNF-α*, and* Mx1*) in CIHKM cells treated with SA are shown in Figure 3.

Expression of* MyD88* gene (Figure 3(a)) in CIHKM cells stimulated with 1 or 10 *μ*g mL^−1^ SA was significantly higher at 8 and 12 h poststimulation (hps) as compared to the control. However, SA had no significant effect on* MyD88* expression at 2 and 24 hps.

Exposure of CIHKM cells to SA at any concentration (1, 10, and 50 *μ*g mL^−1^) for 2 h and 8 h exhibited striking upregulation of* IL-1β* expression (*P* < 0.05). However, no significant induction in* IL-1β* expression was observed at 12 and 24 hps with SA (*P* > 0.05) (Figure 3(b)).

Expression of* TNF-α* is shown in Figure 3(c). Expression of the* TNF-α* gene was significantly higher in CIHKM cells at 2 to 12 hps with SA and highest expression was at 8 hps with 10 *μ*g mL^−1^ of SA (*P* > 0.05). Longer exposure (i.e., 24 hps) had no significant effect on* TNF-α*. Exposure of CIHKM cells to the medium concentration of SA (10 *μ*g mL^−1^) had higher expression than the low or high concentrations.

Exposure of CIHKM cells to SA showed a time-dependent induction of* Mx1* expression as shown in Figure 3(d). Two-hour exposure of CIHKM cells to SA did not significantly affect* Mx1* gene expression. However, higher expression (*P* < 0.05) of* Mx1* gene was detected at 8 and 12 hps with SA, and thereafter, expression was noticeably decreased to a nonsignificant level.

### 3.4. Effect of SA on Immunological Parameters in* C. idella*

**Figure 4:**
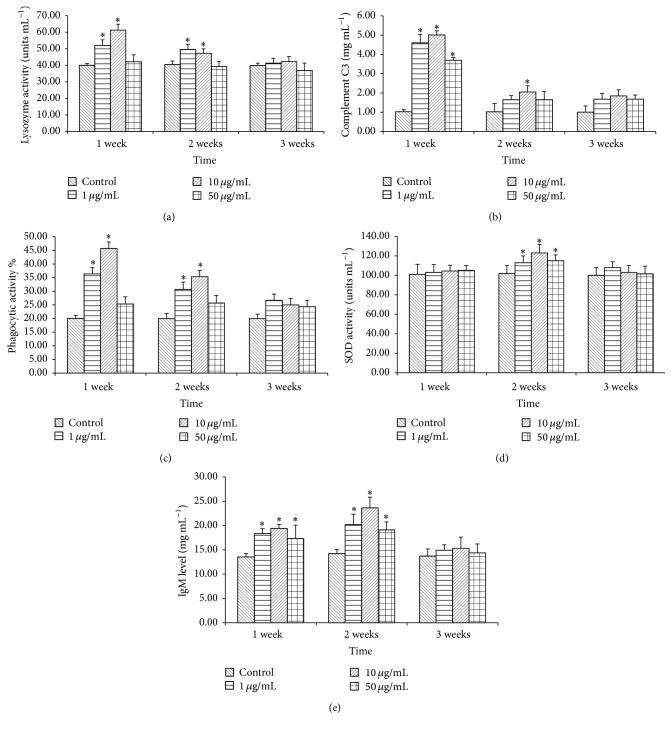
Effects of SA on nonspecial immune responses in* C. idella* immunised (i.p) with SA at different time points. (a) Lysozyme activity; (b) complement C3 level; (c) phagocytic activity; (d) SOD activity; (e) IgM level. Bars represent the mean ± S.E.M (*n* = 9). A significant difference compared to control on the same sampling week is indicated by an asterisk (*∗*).

Results of immunological parameters are shown in Figure 4. A significant increase in serum lysozyme activity was observed in fish after 1-2 weeks of immunisation with 1 or 10 *μ*g mL^−1^ SA, and thereafter it turned to normal level. However, fish immunised with any concentration of SA exhibited no significant differences in lysozyme activity (*P* > 0.05) at 3 weeks after immunisation (Figure 4(a)).

Fish immunised with SA had significantly higher complement C3 activity at 1 week after immunisation. However, at 2 weeks after immunisation, higher (*P* < 0.05) activity was observed in the 10 *μ*g mL^−1^ SA treated group (Figure 4(b)).

The phagocytic activity was significantly higher in fish groups immunised with 1–10 *μ*g mL^−1^ of SA for 1-2 weeks (Figure 4(c)). However, higher concentration of SA had no significant effect on phagocytic activity at any point of time.

SOD activity was higher (*P* < 0.05) in the treated groups only at 2 weeks after injection (Figure 4(d)). However, at 1 or 3 weeks after immunisation, no significant effects on SOD activities were detected.

Serum IgM levels (Figure 4(e)) showed an increasing (*P* < 0.05) trend up to two weeks after immunisation and turned to normal level subsequently in all treated groups. The highest IgM level recorded in a fish group was after 2 weeks of immunisation with 10 *μ*g mL^−1^ of SA.

## 4. Discussion

Plant products as ecological sources of medicine have been used widely for disease control in aquaculture. Several bioactive products from plants, which have potential anthelmintic, antibacterial, antiviral, and even immunostimulatory properties, have been reported in numerous studies [2]. Previously, only the anthelmintic effect of SA against* D. intermedius* in goldfish was documented [11]. However, the present study demonstrated the effects of SA on the expression of immune-related genes in CIHKM cells* in vitro* and on immune responses* in vivo* in order to explore the potential of SA as an immunomodulator in fish.

Innate immune responses are triggered upon pathogen recognition by various types of pattern recognition receptors (PRRs), such as Toll-like receptors (TLRs), which can recognise conserved pathogen-associated molecular patterns (PAMPs) of foreign intruders, including protozoa, bacteria, fungi, and viruses [28, 29]. TLRs operate by initially recognising corresponding PAMPs via extracellular leucine-rich repeat (LRR) motifs, changing this configuration, recruiting Toll/IL-1 receptor (TIR) adaptors including MyD88 to trigger signalling pathways, inducing the production of proinflammatory cytokines, and developing adaptive immunity [30]. After the TLRs recognise specific PAMPs, TLR signalling can be segregated into the* MyD88*-dependent pathway, which causes the activation of the nuclear factor-kappa B (NF-*κ*B) and the expression of proinflammatory genes, such as tumour necrosis factor (TNF) or interleukin-1 (IL-1) [29, 31, 32]. The* MyD88*-independent pathway leads to interferon 3 (IRF3) mediated expression of type I interferons (IFN) and IFN-inducible genes [33]. It was demonstrated that MyD88 played an important role in defense against viral infection and subsequent tissue repair [30]. In the present study, the MyD88 transcript level was found to increase, especially at low concentration (1 *μ*g mL^−1^) at 8 hps. Yu et al. [18] reported that stimulation with two compounds (1,5-anhydro-D-glucitol and 3,4,5-trimethoxy cinnamic acid) isolated from* Polygala tenuifolia* upregulated the expression of the* MyD88* gene in* C. idella* kidney (CIK) cells. Denis and Archambault [34] also reported the induction of* MyD88* expression in CIK cells by secondary metabolites from* Alcaligenes faecalis* FY-3. Moreover, our result was consistent with Su et al. [30] who reported that* MyD88* expression in CIK cells treated with polycytidylic acid was increased at first and then turned to control level at 24 h. These results may be explained by assuming that TLRs can recognise the molecular patterns of SA, or by some other unknown alternative mechanisms related to the immune responses.

The cytokines* IL-1β* and* TNF-α* are produced mainly by monocytes and macrophages, and they regulate several aspects of immune system [35].* IL-1β* can stimulate immune responses by activating lymphocytes or by inducing the release of other cytokines capable of triggering macrophages, NK cells, and lymphocytes [36, 37]. The upregulation of the expression of cytokine-encoding genes by various immunostimulants in fish has been previously reported [34, 36]. Yu et al. [18] demonstrated that active compounds isolated from* P. tenuifolia* stimulated the expression of* IL-1β* in CIK cells. Previous work demonstrated that* IL-1β* was not constitutively expressed in carp,* Cyprinus carpio* L., after stimulation with a bioactive compound [36], and this was in accordance with the results of the present study. Our results demonstrated that 1–10 *μ*g mL^−1^ of SA stimulation for 2–8 h resulted in stronger* IL-1β* expression than that in other experimental groups and at other durations.* TNF-α* works together with* IL-1β* in other cells, which, in turn, produce other cytokines and factors that lead to the activation of the acute phase of the immune response [38].* TNF-α* exerts antiviral effects and may cause direct lysis of tumour cells [38]. In the present study, CIHKM cells stimulated with SA produced significant induction in* TNF-α* expression. Previous studies also reported that stimulation by LPS and flagellin significantly induced the expressions of* TNF-α* in* Oncorhynchus mykiss* [39]. Similar results of enhanced* TNF-α* expression level [18] were also observed in CIK after stimulation with two compounds isolated from* Polygala tenuifolia*.

Mx proteins are key components of the antiviral state, which are interferon (IFN) induced dynamin-like GTPases in many species, and Mx GTPases appear to detect viral infection by sensing nucleocapsid-like structures [40]. Generally, Mx proteins consist of three domains including the C-terminal region that is divergent and acts as an effector domain, which facilitates defense against a diverse range of viruses [14, 40]. The results of our investigation indicated that the* Mx1* gene was significantly induced after 24 h exposure to SA.

The innate immune system is a fundamental defense mechanism in fish [41]. Lysozyme is a vital antibacterial effector molecule, which can be regarded as an opsonin by activating phagocytes and the complement system [42]. The increase in serum lysozyme levels suggests elevation of various humoral factors that can protect the host during pathogen invasion [43]. In the present study, the low and medium concentrations of SA revealed a positive influence on the lysozyme activity at 1 to 2 weeks after immunisation with SA. A previous study [44] also reported that dietary administration of herbal extracts enhanced the lysozyme activity in fish. Several studies have shown that external factors such as nutrients, probiotics, and vitamins influence complement activity in fish [45]. The complement cascade consists of one or a combination of three pathways, namely, the alternate, classical, and lectin pathways [46]. All the three pathways unite at a common amplification step involving complement C3 and proceed through a terminal pathway that leads to the formation of a membrane attack complex, which can lyse pathogens directly [47, 48]. In the present study, serum complement C3 levels were found to be significantly higher in the first week after treatment. Complement activation is usually beneficial to fish, but persistent activation of the complement could cause side effects and immunosuppression in the host [47]. Therefore, short-term activation of serum complement C3 in the present study may provide health benefits to fish.

The present study revealed that phagocytic activity was significantly higher (*P* < 0.05) in fish after 1 to 2 weeks after immunisation with SA. In another study, similar results of higher phagocytic activity were observed in carp after administration of herbal extracts (*A. radix* and* G. lucidum*) [44]. Phagocytosis is an important cellular response in the fish immune system [41], and several studies have confirmed that herbal medicines can enhance phagocytic activity in fish [49, 50]. A recent study suggested that increased* IL-1β* transcripts in the stimulated cells might be a result of enhanced phagocytosis and activation of inflammasomes [48]. Similarly, increased SOD activity was observed in* Labeo rohita* administered herbal extracts [49, 50]. SOD catalyses the dismutation of the highly reactive (^−^O_2_) less reactive H_2_O_2_ and functions in the main antioxidant defense system in response to oxidative stress [51]. Enhanced levels of serum immunoglobulin are thought to be associated with a stronger immune response in fish. In the present study, significant enhancements in serum immunoglobulin level was observed in fish immunised with 1–10 *μ*g mL^−1^ SA for up to 2 weeks. In a recent study, Giri et al. [50] demonstrated that* L. rohita* fed diet supplemented with guava leaf powder exhibited higher serum immunoglobulin activity, which support our result of enhanced serum immunoglobulin after SA stimulation. The increased activities of serum lysozyme, complement C3, PA, SOD, and IgM in the present study indicate that SA plays a significant role in enhancing immune responses in grass carp* C. idella.* Moreover, isolated compound (SA) at low and medium concentrations had no toxicity towards CIHKM cells, and at high concentration, it had a slightly toxic effect on the cells. This noncytotoxic nature of SA (1 to 10 *μ*g mL^−1^) ensured that its influence on cytokine responses was not attributed to the production of secondary stimulation induced by inflammatory mediators released by dead cells.

An experimental infection provides an opportunity to evaluate the effectiveness of SA stimulation in terms of protection against pathogens [46]. In the present study, fish immunised with 10 *μ*g mL^−1^ SA exhibited the highest postchallenge survival (73.33%), followed by those immunised with 1 *μ*g mL^−1^ SA (56.66%). Recent studies have reported that dietary administration of guava leaves or* Chlorophytum borvilianum* polysaccharide enhanced the postchallenge survival of* L. rohita* against* A. hydrophila* infection [49, 50]. The enhanced immune parameters observed in fish immunised with 10 *μ*g mL^−1^ SA might be associated with the elevated resistance of the fish against* A. hydrophila* and the resulting higher postchallenge survival rates.

## 5. Conclusion

In conclusion, the findings of this study demonstrate the immunostimulatory properties of the compound kaempferol 3-a-L-(4-O-acetyl) rhamnopyranoside-7-a-L-rhamnopyranoside (SA) isolated from* D. crassirhizoma*, which increased the immune responses (e.g., lysozyme activity, complement C3, phagocytic activity, SOD activity, and IgM level) of fish* in vivo* and induced expression of immune-related genes (e.g.,* MyD88*,* IL-1β*,* TNF-α*, and* Mx1*)* in vitro*. Further, it enhanced the resistance of fish against pathogen infection. Hence, this natural immunostimulant could be a potential substitute for antibiotics and chemicals in aquaculture practices. However, studies involving use of SA as a feed supplement or oral immunisation on growth promotion and the specific mechanisms controlling immune response modulation are currently under progress in our laboratory.

## Figures and Tables

**Table 1:** Primers used for the analysis of mRNA expression by real-time PCR.

Genes	Primer sequence	Accession number
*β-actin*	F: 5′GATGATGAAATTGCCGCACTG 3′	M25013
	R: 5′ACCGACCATGACGCCCTGATGT 3′	

*IL-1β*	F: 5′GGAGAATGTGATCGAAGAGCGT 3′	EU047716
	R: 5′GCTGATAAACCATCCGGGA 3′	

*TNF-α*	F: 5′TGTGCCGCCGCTGTCTGCTTCACGCT 3′	EU047718
	R: 5′GATGAGGAAAGACACCTGGCTGTAGA 3′	

*MyD88*	F: 5′GAAATGATGGACTTTACCTACCTG 3′	FJ843088
	R: 5′ACATCTTTCCTTTCGGCTTTT 3′	

*Mx1*	F: 5′CTGGGGAGGAAGTAAAGTGTTCT 3′	HQ245104
	R: 5′CAGCATGGATTCTGCCTGG 3′	

**Table 2:** Ingredient and chemical proximate composition of basic diet (% dry matter).

Ingredient	%
Soybean meal	43
Fish meal	35
Wheat meal	15
Corn meal	5
*α*-starch	1
Mineral and vitamin mixture^a^	1

*Proximate analysis*	
Crude protein%	24.8
Crude lipid%	1.9
Ash%	10.8

^a^Every 250 g of mineral-vitamin mixture provided vitamin A, 500,000 IU; vitamin D3, 100,000 IU; vitamin B1, 7 g; vitamin B2, 20 g; vitamin B6, 6 g; vitamin B12, 80 mg; vitamin E, 30 g; vitamin K3, 6 g; vitamin C, 50 g; pantothenate, 15 g; niacin, 65 g; folic acid, 3 g; inositol, 65 g; biotin, 150 mg.

## References

[1] Fisheries and Aquaculture Department (2012). *The State of World Fisheries and Aquaculture*.

[2] Reverter M., Bontemps N., Lecchini D., Banaigs B., Sasal P. (2014). Use of plant extracts in fish aquaculture as an alternative to chemotherapy: current status and future perspectives. *Aquaculture*.

[3] Wang Y.-C., Chang P.-S., Chen H.-Y. (2008). Differential time-series expression of immune-related genes of Pacific white shrimp *Litopenaeus vannamei* in response to dietary inclusion of *β*-1,3-glucan. *Fish & Shellfish Immunology*.

[4] Cabello F. C. (2006). Heavy use of prophylactic antibiotics in aquaculture: a growing problem for human and animal health and for the environment. *Environmental Microbiology*.

[5] Chakraborty S. B., Hancz C. (2011). Application of phytochemicals as immunostimulant, antipathogenic and antistress agents in finfish culture. *Reviews in Aquaculture*.

[6] Citarasu T. (2010). Herbal biomedicines: a new opportunity for aquaculture industry. *Aquaculture International*.

[7] Lee S.-M., Na M.-K., Na R.-B., Min B.-S., Lee H.-K. (2003). Antioxidant activity of two phloroglucinol derivatives from *Dryopteris crassirhizoma*. *Biological and Pharmaceutical Bulletin*.

[8] Mazzio E. A., Soliman K. F. A. (2009). In vitro screening for the tumoricidal properties of international medicinal herbs. *Phytotherapy Research*.

[9] Na M., Jang J., Min B. S. (2006). Fatty acid synthase inhibitory activity of acylphloroglucinols isolated from *Dryopteris crassirhizoma*. *Bioorganic & Medicinal Chemistry Letters*.

[10] Gao Z., Ali Z., Zhao J. (2008). Phytochemical investigation of the rhizomes of *Dryopteris crassirhizoma*. *Phytochemistry Letters*.

[11] Jiang B., Chi C., Fu Y.-W., Zhang Q.-Z., Wang G.-X. (2013). *In vivo* anthelmintic effect of flavonol rhamnosides from *Dryopteris crassirhizoma* against *Dactylogyrus intermedius* in goldfish (*Carassius auratus*). *Parasitology Research*.

[12] Watanuki H., Chakraborty G., Korenaga H., Kono T., Shivappa R. B., Sakai M. (2009). Immunostimulatory effects of natural human interferon-alpha (huIFN-*α*) on carps *Cyprinus carpio* L. *Veterinary Immunology and Immunopathology*.

[13] Aoki T., Takano T., Santos M. D., Kondo H., Hirono I. (2008). Molecular innate immunity in teleost fish: review and future perspectives. *Fisheries for Global Welfare and Environment, Memorial Book of the 5th World Fisheries Congress*.

[14] Zenke K., Kim K. H. (2009). Molecular cloning and expression analysis of three Mx isoforms of rock bream, *Oplegnathus fasciatus*. *Fish & Shellfish Immunology*.

[15] Takeuchi O., Hoshino K., Akira S. (2000). Cutting edge: TLR2-deficient and MyD88-deficient mice are highly susceptible to *Staphylococcus aureus* infection. *The Journal of Immunology*.

[16] Chi C., Jiang B., Yu X.-B., Liu T.-Q., Xia L., Wang G.-X. (2014). Effects of three strains of intestinal autochthonous bacteria and their extracellular products on the immune response and disease resistance of common carp, *Cyprinus carpio*. *Fish & Shellfish Immunology*.

[17] Secombes C. J. (1990). Isolation of salmonid macrophages and analysis of their killing activity. *Techniques in Fish Immunology*.

[18] Yu X.-B., Liu G.-L., Zhu B., Hao K., Ling F., Wang G.-X. (2014). *In vitro* immunocompetence of two compounds isolated from *Polygala tenuifolia* and development of resistance against grass carp reovirus (GCRV) and *Dactylogyrus intermedius* in respective host. *Fish & Shellfish Immunology*.

[19] Livak K. J., Schmittgen T. D. (2001). Analysis of relative gene expression data using real-time quantitative PCR and the 2-ΔΔCT method. *Methods*.

[20] Cheng A.-C., Cheng S.-A., Chen Y.-Y., Chen J.-C. (2009). Effects of temperature change on the innate cellular and humoral immune responses of orange-spotted grouper *Epinephelus coioides* and its susceptibility to *Vibrio alginolyticus*. *Fish and Shellfish Immunology*.

[21] Ellis A. E. (2009). Lysozyme assays. *Techniques in Fish Immunology*.

[22] Li X., Liu L., Zhang Y., Fang Q., Li Y., Li Y. (2013). Toxic effects of chlorpyrifos on lysozyme activities, the contents of complement C3 and IgM, and IgM and complement C3 expressions in common carp (*Cyprinus carpio* L.). *Chemosphere*.

[23] He S., Zhou Z., Liu Y. (2009). Effects of dietary *Saccharomyces cerevisiae* fermentation product (DVAQUA®) on growth performance, intestinal autochthonous bacterial community and non-specific immunity of hybrid tilapia (*Oreochromis niloticus* ♀×*O. aureus* ♂) cultured in cages. *Aquaculture*.

[24] Lothar T. (1999). Clinical laboratory diagnostics. *Clinical Chemistry and Laboratory Medicine*.

[25] Ai Q., Mai K., Tan B. (2006). Effects of dietary vitamin C on survival, growth, and immunity of large yellow croaker, *Pseudosciaena crocea*. *Aquaculture*.

[26] Chi C., Liu J.-Y., Fei S.-Z. (2014). Effect of intestinal autochthonous probiotics isolated from the gut ofsea cucumber (*Apostichopus japonicus*) on immune response andgrowth of *A. japonicus*. *Fish and Shellfish Immunology*.

[27] Jiang H.-F., Liu X.-L., Chang Y.-Q., Liu M.-T., Wang G.-X. (2013). Effects of dietary supplementation of probiotic *Shewanella colwelliana* WA64, *Shewanella olleyana* WA65 on the innate immunity and disease resistance of abalone, *Haliotis discus hannai* Ino. *Fish & Shellfish Immunology*.

[28] Takeda K., Akira S. (2005). Toll-like receptors in innate immunity. *International Immunology*.

[29] Yao C.-L., Kong P., Wang Z.-Y. (2009). Molecular cloning and expression of MyD88 in large yellow croaker, *Pseudosciaena crocea*. *Fish & Shellfish Immunology*.

[30] Su J., Dong J., Huang T., Zhang R., Yang C., Heng J. (2011). Myeloid differentiation factor 88 gene is involved in antiviral immunity in grass carp *Ctenopharyngodon idella*. *Journal of Fish Biology*.

[31] Medzhitov R., Preston-Hurlburt P., Kopp E. (1998). MyD88 is an adaptor protein in the hToll/IL-1 receptor family signaling pathways. *Molecular Cell*.

[32] Janeway C. A., Medzhitov R. (2002). Innate immune recognition. *Annual Review of Immunology*.

[33] Kawai T., Takeuchi O., Fujita T. (2001). Lipopolysaccharide stimulates the MyD88-independent pathway and results in activation of IFN-regulatory factor 3 and the expression of a subset of lipopolysaccharide-inducible genes. *The Journal of Immunology*.

[34] Denis F., Archambault D. (2001). Molecular cloning and characterization of beluga whale (*Delphinapterus leucas*) interleukin-1beta and tumor necrosis factor-alpha. *Canadian Journal of Veterinary Research*.

[35] Yuan C., Pan X., Gong Y. (2008). Effects of Astragalus polysaccharides (APS) on the expression of immune response genes in head kidney, gill and spleen of the common carp, *Cyprinus carpio* L. *International Immunopharmacology*.

[36] Low C., Wadsworth S., Burrells C., Secombes C. J. (2003). Expression of immune genes in turbot (*Scophthalmus maximus*) fed a nucleotide-supplemented diet. *Aquaculture*.

[37] Luster M. I., Simeonova P. P., Gallucci R., Matheson J. (1999). Tumor necrosis factor *α* and toxicology. *CRC Critical Reviews in Toxicology*.

[38] Chettri J. K., Raida M. K., Holten-Andersen L., Kania P. W., Buchmann K. (2011). PAMP induced expression of immune relevant genes in head kidney leukocytes of rainbow trout (*Oncorhynchus mykiss*). *Developmental and Comparative Immunology*.

[39] Peng L., Yang C., Su J. (2012). Protective roles of grass carp *Ctenopharyngodon idella* Mx isoforms against grass carp reovirus. *PLoS ONE*.

[40] Magnadóttir B. (2006). Innate immunity of fish (overview). *Fish & Shellfish Immunology*.

[41] Wang G.-X., Liu Y.-T., Li F.-Y., Gao H.-T., Lei Y., Liu X.-L. (2010). Immunostimulatory activities of *Bacillus simplex* DR-834 to carp (*Cyprinus carpio*). *Fish and Shellfish Immunology*.

[42] Harikrishnan R., Balasundaram C., Heo M.-S. (2010). Effect of probiotics enriched diet on *Paralichthys olivaceus* infected with lymphocystis disease virus (LCDV). *Fish & Shellfish Immunology*.

[43] Yin G., Ardó L., Thompson K. D., Adams A., Jeney Z., Jeney G. (2009). Chinese herbs *(Astragalus radix* and *Ganoderma lucidum*) enhance immune response of carp, *Cyprinus carpio*, and protection against *Aeromonas hydrophila*. *Fish & Shellfish Immunology*.

[44] Giri S. S., Sukumaran V., Sen S. S., Jena P. K. (2014). Effects of dietary supplementation of potential probiotic *Bacillus subtilis* VSG1 singularly or in combination with *Lactobacillus plantarum* VSG3 or/and *Pseudomonas aeruginosa* VSG2 on the growth, immunity and disease resistance of *Labeo rohita*. *Aquaculture Nutrition*.

[45] Giri S. S., Sen S. S., Sukumaran V. (2012). Effects of dietary supplementation of potential probiotic *Pseudomonas aeruginosa* VSG-2 on the innate immunity and disease resistance of tropical freshwater fish, *Labeo rohita*. *Fish and Shellfish Immunology*.

[46] Sun Y.-Z., Yang H.-L., Ma R.-L., Lin W.-Y. (2010). Probiotic applications of two dominant gut *Bacillus* strains with antagonistic activity improved the growth performance and immune responses of grouper *Epinephelus coioides*. *Fish & Shellfish Immunology*.

[47] Boshra H., Li J., Sunyer J. O. (2006). Recent advances on the complement system of teleost fish. *Fish & Shellfish Immunology*.

[48] Giri S. S., Sen S. S., Chi C. (2015). *Chlorophytum borivilianum* polysaccharide fraction provokes the immune function and disease resistance of *Labeo rohita* against *Aeromonas hydrophila*. *Journal of Immunology Research*.

[49] Bilen S., Biswas G., Otsuyama S., Kono T., Sakai M., Hikima J.-I. (2014). Inflammatory responses in the Japanese pufferfish (*Takifugu rubripes*) head kidney cells stimulated with an inflammasome-inducing agent, nigericin. *Developmental & Comparative Immunology*.

[50] Giri S. S., Sen S. S., Chi C. (2015). Effect of guava leaves on the growth performance and cytokine gene expression of *Labeo rohita* and its susceptibility to *Aeromonas hydrophila* infection. *Fish and Shellfish Immunology*.

[51] Zhao Y., Zhang W., Xu W., Mai K., Zhang Y., Liufu Z. (2012). Effects of potential probiotic *Bacillus subtilis* T13 on growth, immunity and disease resistance against *Vibrio splendidus* infection in juvenile sea cucumber *Apostichopus japonicus*. *Fish & Shellfish Immunology*.

